# Histology-Specific Treatment Strategies and Survival Prediction in Lung Cancer Patients with Spinal Metastases: A Nationwide Analysis

**DOI:** 10.3390/cancers17081374

**Published:** 2025-04-21

**Authors:** Abdul Karim Ghaith, Xinlan Yang, Taha Khalilullah, Xihang Wang, Melanie Alfonzo Horowitz, Jawad Khalifeh, A. Karim Ahmed, Tej Azad, Joshua Weinberg, Abdel-Hameed Al-Mistarehi, Chase Foster, Meghana Bhimreddy, Arjun K. Menta, Kristin J. Redmond, Nicholas Theodore, Daniel Lubelski

**Affiliations:** 1 Department of Neurosurgery, School of Medicine, Johns Hopkins University, Baltimore, MD 21205, USA; aghaith1@jh.edu (A.K.G.); xyang118@jhmi.edu (X.Y.); tkhalil1@jh.edu (T.K.); xwang404@jhmi.edu (X.W.); malfonz1@jhu.edu (M.A.H.); jkhalif1@jhmi.edu (J.K.); aahmed33@jhmi.edu (A.K.A.); tazad1@jhmi.edu (T.A.); aalmist1@jh.edu (A.-H.A.-M.); mbhimre1@jhmi.edu (M.B.); arjun.menta@jhmi.edu (A.K.M.); kjanson3@jhmi.edu (K.J.R.); theodore@jhmi.edu (N.T.); 2 Department of Neurosurgery, School of Medicine, Ohio State University, Columbus, OH 43210, USA; joshua.weinberg@osumc.edu; 3 Department of Neurosurgery, School of Medicine, George Washington University, Washington, DC 20052, USA; cfoste43@jh.edu

**Keywords:** lung cancer, spine metastases, NSCLC, SCLC, radiation therapy, mortality risk prediction

## Abstract

Background/Objectives: Spinal metastases are a common and severe complication of lung cancer, particularly in small cell lung cancer (SCLC), and are associated with poor survival. Despite advancements in treatment, optimal management strategies remain unclear, with significant differences between non-small cell lung cancer (NSCLC) and SCLC. This study evaluates treatment patterns, survival outcomes, and prognostic factors in lung cancer patients with spinal metastases, integrating deep learning survival prediction models. Methods: This retrospective cohort study analyzed the National Cancer Database (NCDB) to identify NSCLC and SCLC patients diagnosed with spinal metastases. Demographics and treatment modalities were analyzed and adjusted for age, sex, and comorbidities. Kaplan–Meier analysis and Cox proportional hazards models assessed overall survival (OS). Five advanced survival prediction models estimated 1-year and 10-year mortality, with feature importance determined via permutation analysis. Results: Among 428,919 lung cancer patients, 5.1% developed spinal metastases, with a significantly higher incidence in SCLC (13.6%) than in NSCLC (5.1%). SCLC patients had poorer OS. Radiation therapy alone was the predominant treatment, and stereotactic body radiation therapy (SBRT) predicted better short- and long-term survival compared to other radiation techniques. High-dose radiation (71–150 Gy BED) improved OS in NSCLC, while reirradiation benefited NSCLC but had a limited impact in SCLC. SurvTrace demonstrated the highest predictive accuracy for 1-year and 10-year mortality, identifying age, radiation dose, reirradiation, and race as key prognostic factors. Conclusions: The management of spinal metastases requires a histology-specific approach. Radiation remains the primary treatment, with SBRT predicting better short- and long-term survival. High-dose radiation and reirradiation should be considered for NSCLC, while the benefits are limited in SCLC. These findings support histology-specific treatment strategies to improve survival of patients with metastatic lung cancer to the spine.

## Simple Summary

Lung cancer frequently spreads to the spine, leading to severe complications and reduced survival. Small cell lung cancer patients are particularly affected, yet optimal treatment strategies for spinal metastases remain unclear. This study examines treatment patterns, survival outcomes, and predictive factors using a large national dataset. Findings reveal that radiation therapy is the most common treatment, with higher radiation doses and reirradiation improving survival in non-small cell lung cancer but having limited effects in small cell lung cancer. Stereotactic body radiation therapy or surgery predicted better short-term and long-term survival. Machine learning models identified advanced age, radiation dose, reirradiation, and race as key prognostic factors. These insights provide guidance to clinicians in optimizing therapy for lung cancer patients with spinal metastases.

## 1. Introduction

Lung cancer is one of the leading causes of cancer-related mortality worldwide, accounting for approximately 1.76 million deaths annually [1]. A hallmark of advanced lung cancer is its high propensity for skeletal metastasis, with the spine being the most commonly involved site, affecting up to 40% of patients [2]. Histologically, lung cancer is broadly categorized into two major types: non-small cell lung cancer (NSCLC) and small cell lung cancer (SCLC). NSCLC generally carries a better prognosis, with 5- and 10-year survival rates of 10% and 6%, respectively, whereas SCLC is associated with more aggressive behavior and lower 5- and 10-year survival rates of 5% and 2% [3]. The prognosis becomes especially poor when patients with primary lung cancer develop spinal metastases, with median survival often reported to be less than six months [4]. These patients frequently experience serious complications, including intractable back pain, spinal instability, and neurological deficits due to spinal cord compression, conditions that severely impair functional independence and quality of life [5,6].

Surgical intervention remains the primary treatment of spinal metastases, often involving decompression and stabilization. Radiation therapy (RT), whether used alone or as an adjunct to surgery, provides additional symptom relief and modest survival benefits. However, radiation dosing is frequently limited by spinal cord tolerance, posing a challenge in achieving optimal control [7]. Despite the evolution of minimally invasive surgical techniques and advancements in high-dose, image-guided radiation delivery, the optimal management approach remains controversial [8,9,10]. Moreover, limited data exist comparing outcomes between NSCLC and SCLC patients with spinal metastases, particularly in terms of how tumor histology influences treatment efficacy and survival. These gaps limit the development of histology-specific treatment strategies for this high-risk population.

To address these limitations, our study uses a large, nationally representative cohort from the National Cancer Database (NCDB) to achieve the following objectives: to compare treatment patterns and clinical outcomes in patients with spinal metastases originating from NSCLC versus SCLC; to identify histology-specific prognostic factors associated with long-term survival; and to implement and evaluate deep learning-based survival models to enhance outcome prediction in this complex patient population.

## 2. Materials and Methods

### 2.1. Cohort Selection

The study cohort was queried from the National Cancer Database (NCDB) between 2004 and 2018, from which we identified patients with histologically confirmed primary lung cancer. Based on the histology, the cohort was then stratified into two cohorts: small-cell lung cancer (SCLC) and non-small cell lung cancer (NSCLC), while ones with benign tumors were excluded. The study followed Strengthening the Reporting of Observational Studies in Epidemiology (STROBE) guidelines for cohort studies. Given the use of de-identified data, this study was exempt from formal Institutional Review Board approval and informed consent requirements under the Health Insurance Portability and Accountability Act (HIPAA).

### 2.2. Patient Demographics and Disease and Treatment Characteristics

Patients’ age, sex, race, and Charlson–Deyo Comorbidity Classification (CDCC) scores were documented. The size of the primary lung tumor was recorded as the largest diameter in millimeters. Primary treatments to the lungs and secondary treatments directed at spinal metastases were specified. Spine surgery was defined as non-primary procedures targeting vertebral metastases. Radiation therapy was defined as any treatment targeting the vertebral column and was further stratified by beam technology and biologically effective dose (BED). Beam types included external beam radiation therapy (EBRT), intensity-modulated radiation therapy (IMRT), three-dimensional conformal radiation therapy (3D-CRT), and stereotactic body radiation therapy or stereotactic radiosurgery (SBRT/SRS). BED was calculated using the linear-quadratic model: BED = n × d × (1 + d/(α/β)), where n is the number of fractions, d is the dose per fraction, and the α/β ratio was assumed to be 10 Gy for tumor tissue. BED values were stratified into three categories: low-dose (≤70 Gy), intermediate-dose (71–150 Gy), and high-dose (>150 Gy). Reirradiation was defined as the receipt of two distinct courses of spine-directed radiation [11]. Chemotherapy at any point during the treatment course was recorded.

### 2.3. Primary and Secondary Outcomes

The primary outcome was long-term (10 or more years) overall survival (OS). Secondary outcomes included length of stay (LOS), 30-day readmission rate, and short-term mortality rate at 1 year.

### 2.4. Statistical Analysis

Categorical variables were summarized as frequencies and percentages and analyzed using chi-square or Fisher’s exact tests. Continuous variables were represented as means with standard deviations (SDs) and assessed using independent samples *t*-tests for normal distributions or Mann–Whitney U tests for non-normal distributions. A significance level of *p* < 0.05 was applied. KM survival analysis with log-rank tests was performed to evaluate overall survival (OS). Histology-stratified KM survival analyses were also performed to explore the differential impact of factors on OS in SCLC and NSCLC. K-Nearest Neighbors matching was performed at a 1:1 ratio between NSCLC and SCLC, adjusting for age groups (pediatric: ≤21 years; adult: >21 years), sex, and CDCC comorbidity scores within both the entire population and spine metastasis cohort. The effectiveness of the KNN propensity score matching was evaluated by post-matching covariate balance, assessed using Standardized Mean Differences (SMDs), with all covariates demonstrating adequate balance (SMD < 0.1). The selection of matching variables—age group, sex, and CDCC comorbidity score—was based on clinical relevance and data availability. These covariates are widely recognized as significant confounders in survival analyses and influence both treatment selection and prognosis in lung cancer patients.

To evaluate and identify independent prognostic factors of long-term survival, we added a multivariable Cox proportional hazards model to quantify the relative impact of clinical, demographic, and treatment-related variables in both the overall cohort and the subset of patients with spinal metastases while adjusting for potential confounders.

### 2.5. Deep Learning Implementation

Data preprocessing utilized the MissForest algorithm for handling missing data across multiple variable types, with dataset partitioning into 80% training and 20% testing sets. Class imbalance in survival outcomes was addressed through the Synthetic Minority Over-sampling Technique (SMOTE), followed by variable normalization and standardization to ensure consistent model training [12]. Initial data preprocessing involved comprehensive cleaning and standardization steps, with missing values handled through the MissForest algorithm. To address missing values, we applied the MissForest algorithm, a non-parametric imputation method based on Random Forests. The algorithm was configured with “n_estimators = 100 trees” and a maximum of 10 iterations (max_iter = 10). The stopping criterion was based on the minimal change in normalized root mean squared error (NRMSE) between iterations. Across the dataset, less than 5% of values were missing per variable. The algorithm converged within 6 iterations with stable NRMSE, indicating effective imputation without overfitting or data distortion.

Mortality prediction models and their temporal performance were evaluated through longitudinal area under the curve (AUC) analysis over a 12-month then 120-month follow-up period in patients diagnosed with lung cancer with spine metastasis and received radiation therapy. The predictive modeling approach incorporated multiple machine learning methodologies, including Random Survival Forest, CoxPH, SurvTrace, Gradient Boosting, and DeepSurv. Continuous variables underwent z-score normalization ((x − μ)/σ), while categorical variables were processed using one-hot encoding. Cross-validation employed a nested structure, with an outer 5-fold cross-validation for performance estimation and an inner 3-fold cross-validation for hyperparameter tuning. Model performance was first evaluated using this nested framework on the NCDB dataset, followed by testing on a held-out 20% internal test set. To assess generalizability, external validation was subsequently conducted using an independent cohort from the SEER database.

Model performance was evaluated through time-dependent AUC calculations, with bootstrap resampling (1000 iterations) for 95% confidence intervals, along with a time-dependent concordance index. DeLong’s test assessed the statistical significance of AUC differences with Bonferroni correction (α = 0.05/15). We conducted feature importance analyses. To interpret the predictions of our best-performing model, we employed the SHapley Additive exPlanations (SHAP) approach that assigns an importance value to each feature by assessing its contribution to model outputs. The mean absolute SHAP values were used to rank features. All analyses were conducted using Python 3.8.12 with scikit-learn (0.24.2), PyTorch (1.9.0), and lifelines (0.26.0), adhering to STROBE reporting guidelines for observational studies.

## 3. Results

### 3.1. Cohort Selection, Patient Demographics, and Disease Characteristics

**Figure 1:**
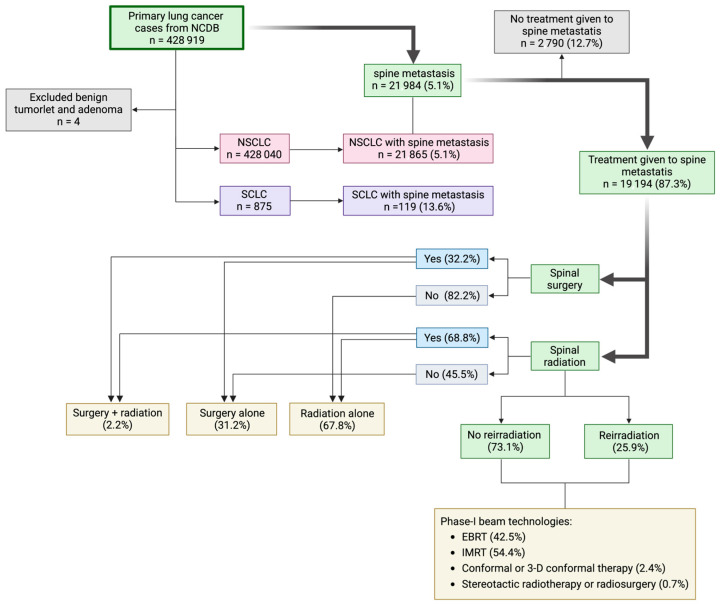
Flowchart of cohort selection and treatment allocation.

The NCDB cohort included 428,919 primary lung cancer patients, with 428,808 classified as non-small cell lung cancer (NSCLC) and 875 as small cell lung cancer (SCLC) (Figure 1). Among these patients, spine metastases occurred in 5.1% of the total population, with varied incidence between subtypes: 5.1% in NSCLC cases and 13.6% in SCLC cases.

Baseline demographic characteristics differed significantly between NSCLC and SCLC groups (Table 1). Before matching, SCLC patients were significantly older on aver-age and had a higher proportion of male patients. White patients were more prevalent in the SCLC group (89.1% vs. 82.7%), whereas Black, Asian, and Hispanic patients were more commonly represented in the NSCLC group (*p* < 0.001). These demographic differences were not only statistically significant but also clinically relevant. In multivariable Cox regression analysis, male sex was associated with a slightly but significantly increased risk of mortality. Moreover, compared to Asian patients, Black patients had significantly higher mortality risk in both the overall and spine metastasis cohorts, while Hispanic and White patients also showed modest but statistically significant risk elevations. Advanced age emerged as the strongest and most consistent predictor of mortality across all models, further confirmed by both Cox regression and machine learning-based feature importance analyses. After matching, differences in racial distribution persisted (*p* = 0.002). Within the spine metastasis population, demographic patterns were similar to the overall population (Table 2). SCLC patients also presented with a significantly higher mean age before matching and a greater prevalence of White patients before and after matching (87.3% vs. 67.3%, *p* < 0.001) compared to NSCLC. Patients with more comorbidities (CDCC scores ≥ 1) were also more prevalent in SCLC before matching (*p* = 0.002). Tumor size remained comparable between groups before and after matching.

### 3.2. Treatment Characteristics

Treatments varied significantly between NSCLC and SCLC. Within the overall lung cancer population, definitive surgery was significantly more frequent in the NSCLC group (56.6% vs. 42.6%, *p* < 0.001), while radiation was significantly more frequent in the SCLC group (64.5% vs. 58.5%, *p* < 0.001) (Table 1). Chemotherapy was also more commonly used in the SCLC group (69.8% vs. 41.0%, *p* < 0.001), with multi-agent regimens being remarkably more prevalent in SCLC compared to NSCLC (60.4% vs. 14.1%, *p* < 0.001) in contrast to single-agent regimens, which were more common in NSCLC (23.1% vs. 4.9%, *p* < 0.001).

Within the spine metastasis population, most of the spine metastases (87.3%) were managed via local therapy, and NSCLC patients more frequently received treatment for their spine metastases compared to the SCLC group both before (87.4% vs. 70.6%, *p* < 0.001) and after matching (84.9% vs. 70.6%, *p* = 0.008) (Table 2). Specifically, radiation alone was the predominant approach in both groups, with a higher frequency in SCLC (90.5% vs. 67.7%, *p* < 0.001). Conversely, surgery alone was more commonly employed in NSCLC cases (31.3% vs. 7.1%, *p* < 0.001). After matching, similar patterns persist with statistical significance reached. Combined surgery and radiation were infrequent across both groups, representing only 1–3% of spine metastasis cases before and after matching.

Among patients who received radiation to the spine metastases, external beam radiation therapy (EBRT) was the most common beam technology in both groups, particularly in SCLC (52.6% vs. 42.5%); in contrast, intensity-modulated radiation therapy (IMRT) was more frequent in NSCLC (54.5% vs. 41%, *p* = 0.023) before matching. However, the difference was no longer significant after matching. Conformal (3-D) therapy and stereotactic body radiation therapy or stereotactic radiosurgery (SBRT/SRS) were relatively rare in both groups. The NSCLC group received significantly higher BED, with 71–150 Gy regimen being the most common, while more SCLC patients received a lower BED (*p* = 0.019), ranging below 70 Gy (*p* < 0.001). However, statistical differences diminished after adjusting for baseline characteristics. Additionally, 26.2% of radiated patients underwent reirradiation, with no differences observed between NSCLC and SCLC.

### 3.3. Clinical Outcomes and Long-Term OS

The hospital length of stay (LOS) differed between groups in the overall lung cancer population, with NSCLC patients showing marginally longer hospitalizations before matching (4.1 ± 8.9 days vs. 3.1 ± 7.3 days, *p* = 0.036), though this difference disappeared after matching (*p* = 0.861) (Table 1). Thirty-day readmission rates remained consistent between groups before and after matching, occurring in approximately 3% of all patients. Palliative care utilization was significantly higher in SCLC patients both before (5.4% vs. 2.2%, *p* < 0.001) and after matching (5.4% vs. 3.0%, *p* = 0.012). While SCLC patients demonstrated a higher frequency of spine metastasis before matching (13.6% vs. 5.1%, *p* < 0.001), this difference became non-significant after matching (13.6% vs. 11.1%, *p* = 0.11). Mortality rates consistently remained higher in SCLC compared to NSCLC, both before and after matching, at all measured timepoints: 1 year (32.7% vs. 19.7%, *p* < 0.001), 5 years (61% vs. 45.6%, *p* < 0.001), 10 years (65.9% vs. 52.8%, *p* < 0.001), and at final follow-up (66.3% vs. 54.1%, *p* < 0.001). In patients with spine metastases, hospital LOS was comparable between NSCLC and SCLC groups, averaging 5.7 days before matching and 3.4 days after matching (Table 2). Both groups demonstrated similarly low 30-day readmission rates. While palliative care utilization was initially higher in SCLC patients before matching (12.6% vs. 6.8%, *p* = 0.012), this difference became non-significant after matching.

**Figure 2:**
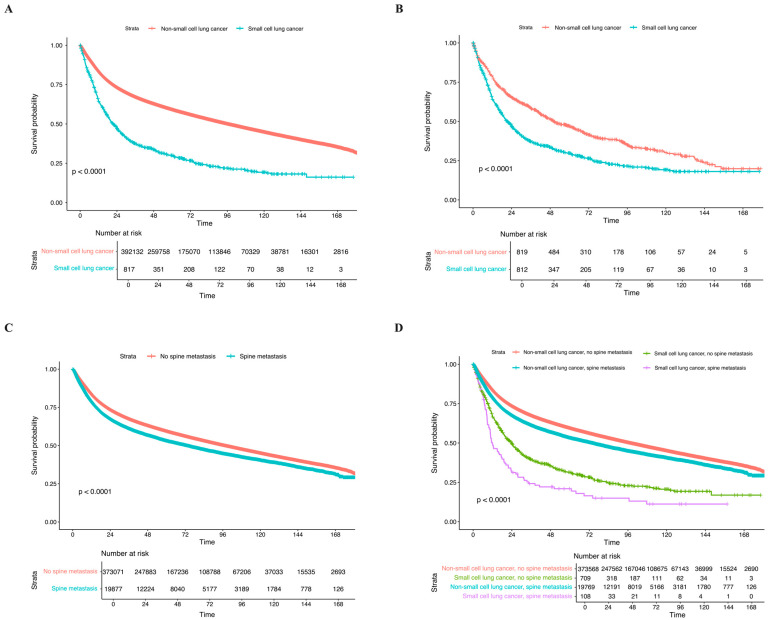
Kaplan–Meier survival analysis of the impact of (**A**) histology; (**B**) histology after matching; (**C**) status of spine metastasis, and (**D**) histology-stratified status of spine metastasis on the long-term OS within the overall population.

**Figure 3:**
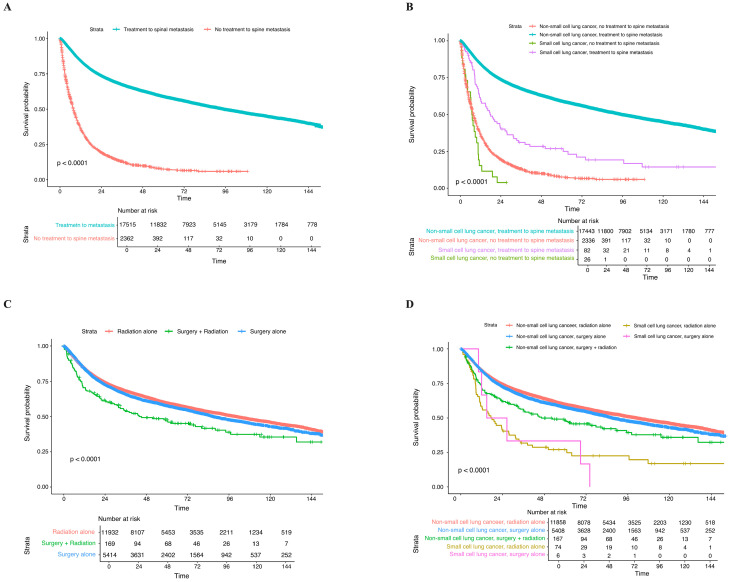
Kaplan–Meier survival analysis of the impact of (**A**) spinal treatment status; (**B**) histology-stratified spinal treatment status with the population with spine metastases; (**C**) spinal treatments; and (**D**) histology-stratified spinal treatments within the population with treated spine metastases.

KM survival analysis demonstrates the impact of factors on the long-term OS. Overall, NSCLC demonstrated a significantly better OS compared to SCLC before matching (*p* < 0.0001) (Figure 2A). This difference persisted after adjusting for age, sex, and comorbidities (*p* < 0.0001) (Figure 2B). Patients with spine metastasis had significantly poorer OS compared to those who did not (*p* < 0.0001); specifically, NSCLC with no spine metastases had the best OS, whereas SCLC patients with spine metastases demonstrated significantly poorer OS (Figure 2C,D). Within the spine metastasis population, patients who received treatments for spine metastases demonstrated significantly better OS than their no-treatment counterparts (*p* < 0.0001) (Figure 3A). Similarly, in the histology-stratified analysis, NSCLC receiving treatment had the best OS, while SCLC without treatment had the poorest OS (*p* < 0.0001) (Figure 3B). In terms of treatments, radiation alone conferred the most survival benefits than surgery alone and combined surgery with radiation (*p* < 0.0001) (Figure 3C). The impact of these treatment approaches was consistent in the NSCLC group when stratified by histology: radiation alone demonstrated better OS compared to SCLC treated with surgery alone; nevertheless, the low number of cases restrained the statistical power (*p* < 0.0001) (Figure 3D).

**Figure 4:**
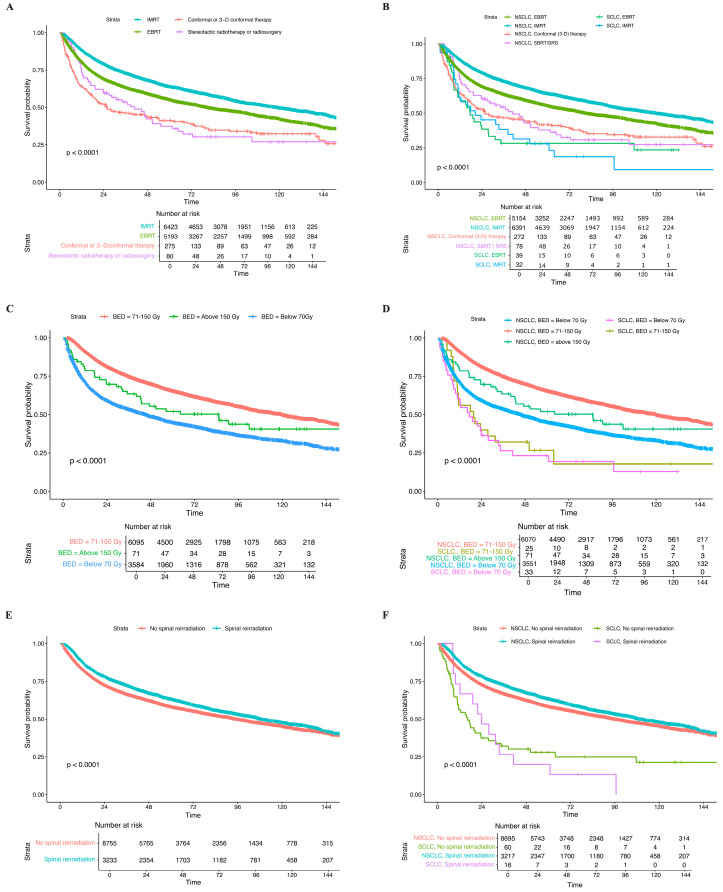
Kaplan–Meier survival analysis of the impact of (**A**) beam technologies; (**B**) histology-stratified beam technologies; (**C**) BED dose strata; (**D**) histology-stratified BED dose strata; (**E**) status of reirradiation; (**F**) histology-stratified status of reirradiation within the spine metastasis population treated with radiation.

In the treatment of spinal metastases with radiation therapy, IMRT demonstrated the highest OS, followed by EBRT. SBRT/SRS provided significant short-term survival benefits within two years compared to conformal (3-D) therapy. However, the OS benefits associated with SBRT/SRS declined beyond two years (*p* < 0.0001) (Figure 4A). When stratified by histology, NSCLC treated with IMRT showed the best OS, and SCLC treated with IMRT had poor OS (*p* < 0.0001) (Figure 4B). A radiation BED of 71–150 Gy demonstrated better OS compared to the other two dose strata, particularly in NSCLC; however, SCLC treated with 71–150 Gy had inferior OS (Figure 4C,D). Reirradiation was generally linked to improved OS (*p* < 0.0001) (Figure 4E). To be specific, NSCLC treated with irradiation had the best OS, whereas SCLC patients undergoing reirradiation showed the poorest OS (*p* < 0.0100) (Figure 4F).

### 3.4. Mortality Risk Prediction

**Figure 5:**
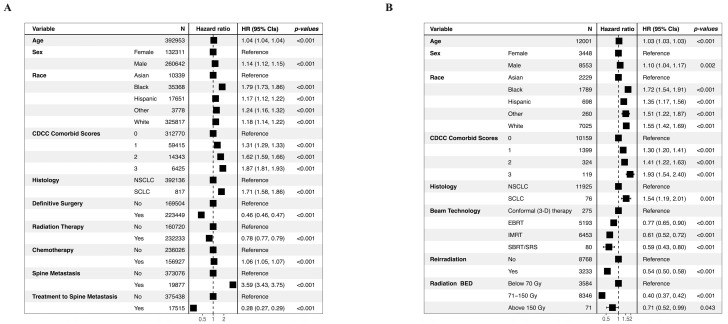
Cox proportional hazards models within (**A**) the overall population; (**B**) the subpopulation of patients with spine metastases treated with radiation.

The Cox proportional hazard model identified several risk factors associated with the entire lung tumor population and the subpopulation of patients with spine metastases receiving radiation to the spine. Male patients exhibited a slightly but significantly higher risk compared to female patients within both populations (Figure 5A,B). Compared to Asian patients, Black patients demonstrated a significantly elevated risk in both populations, while Hispanic and White racial groups also had slightly higher significant mortality risks. The comorbidities scores were positively correlated with the morality risk in both populations. Histologically, SCLC was a significant risk factor in both overall (HR = 1.71, *p* < 0.001) and radiated spine metastases (HR = 1.54, *p* < 0.001) populations.

Within the overall lung cancer population, definitive surgery (HR:0.46, *p* < 0.001) and radiation therapy (HR:0.78, *p* < 0.001) was associated with lower risk; in contrast, chemotherapy was associated with a slightly higher risk (HR:1.06, *p* < 0.001) (Figure 5A). Spine metastasis was associated with a remarkably higher risk of mortality (HR: 3.59, *p* < 0.001); however, active treatments for spine metastasis were associated with lowered risk compared to the no-treatment counterparts (HR: 0.28, *p* < 0.001). Within the subpopulation with radiated spine metastases, radiation parameters were important factors in predicting mortality risk (Figure 5B). Compared to conventional conformal therapy, EBRT (HR: 0.77, *p* < 0.001), IMRT (HR: 0.61, *p* < 0.001), and SBRT/SRS (HR: 0.59, *p* < 0.001) were all associated with lowered risk (Figure 5B). Reirradiation also predicted a lower risk compared to the no-reirradiation counterparts (HR: 0.54, *p* < 0.001). A dose of BED 71–150 Gy was associated with a significantly lowered risk (HR: 0.4, *p* < 0.001), followed by a BED dose above 150 Gy (HR: 0.71, *p* < 0.043).

### 3.5. Feature Importance Analysis

**Figure 6:**
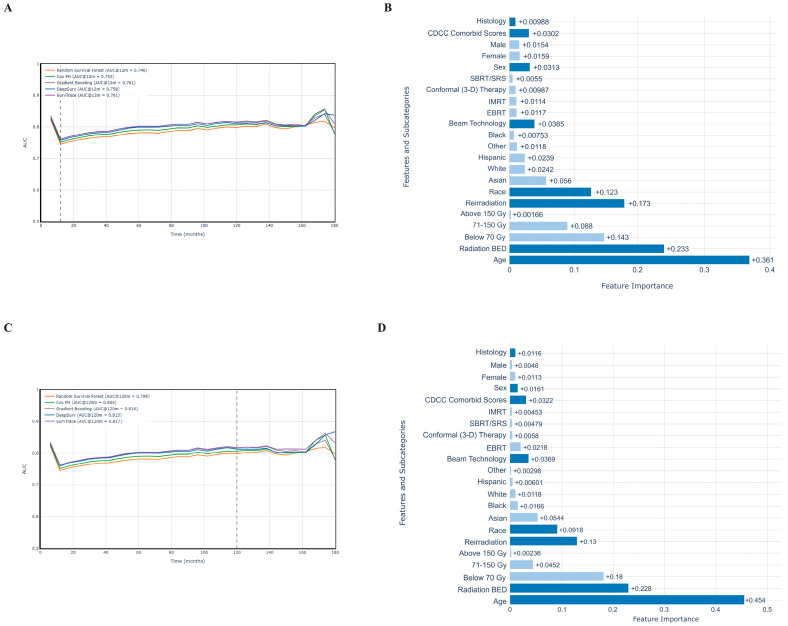
Evaluation of deep learning model performance in prediction of mortality risk at (**A**) 1 year and (**C**) 10 years in patients radiated for spine metastases; SHAP feature importance analysis for factors predicting mortality risk for (**B**) 1 year and (**D**) 10 years.

In the prediction of short-term (1-year) mortality in patients with spinal metastases treated with radiation, the SurvTrace model demonstrated the highest predictive accuracy by showing the largest AUC of 0.761 (Figure 6A). The permutation feature importance analysis based on SurvTrace identified age as the strongest predictive factor, with higher age predicting higher mortality (+0.361). Then, radiation BED strata emerged as the next most important factor, where a low dose below 70 Gy was strongly associated with higher mortality (+0.143). Then, reirradiation of spine metastasis (+0.173), followed by the race group, with Black (+0.00753) predicting lower mortality and Asian (+0.056) predicting higher mortality. Then, beam technologies contributed with a smaller influence, as stereotactic radiotherapy (+0.0055) predicted the lowest mortality. Sex showed a weaker association, with a slight contribution to mortality risk (+0.0313), followed by the comorbidities (CDCC score, +0.0302) and, finally, the metastasis tumor histology (+0.00988) (Figure 6B).

In the prediction of long-term (10-year) mortality in patients with spinal metastases treated with radiation, the SurvTrace model also demonstrated the highest predictive accuracy, achieving an AUC of 0.761 (Figure 6C). The permutation feature importance analysis based on SurvTrace identified age as the strongest predictive factor, with higher age predicting higher mortality (+0.454). Then, radiation BED strata emerged as the next most important factor (+0.228), where a low dose below 70 Gy was strongly associated with higher mortality. Then, the reirradiation of the spinal metastasis followed with a feature importance of +0.13. Race groups contributed to mortality variation, with Hispanic (+0.006) predicting lower mortality and Asian (+0.0544) predicting higher mortality. Then, beam technologies contributed with a smaller influence, as stereotactic radiotherapy (+0.00479) then IMRT (+0.00453) predicted the lowest mortality. Then, sex showed a weaker association, with a slight contribution to mortality risk (+0.0161), followed by comorbidities (CDCC score, +0.0322) and, finally, metastasis tumor histology (+0.0116) (Figure 6D).

## 4. Discussion

In this nationwide cohort, we observed significant histology-specific differences in treatment strategies and survival outcomes among lung cancer patients with spinal metastases. Radiation therapy should be prioritized for managing spinal metastases. SBRT/SRS was associated with better early survival compared to other modalities, while both SBRT/SRS and IMRT demonstrated comparable advantages in long-term survival. High-dose radiation should be utilized when feasible, particularly in NSCLC patients. Reirradiation was linked to improved survival in NSCLC patients with durable life expectancy, whereas similar benefits were not observed in the SCLC cohort. Additionally, machine learning analysis identified age, radiation dose, reirradiation, and race as key predictors of mortality.

Our study provides a comprehensive comparison of NSCLC and SCLC patients with spinal metastases, highlighting key differences in baseline characteristics, treatment patterns, and clinical outcomes. SCLC was associated with a significantly poorer OS and a higher prevalence of spinal metastases compared to NSCLC. Notably, SCLC patients were less likely to receive spine-directed treatments, which our findings identified as critical for improving survival in this population. While aggressive treatment strategies combining surgery and radiation offered limited survival benefits, the use of either surgery or radiation alone, particularly with advanced modalities such as IMRT, demonstrated significant improvements in OS. These findings highlight the importance of spine-directed therapies created for the distinct characteristics of lung cancer histology. They also emphasize the pressing need for advancing therapeutic technologies in order to better manage spinal metastases in patients with primary lung cancer.

SCLC demonstrated poorer outcomes and a higher prevalence of spinal metastases compared to NSCLC in our study, findings that are well supported by the existing literature [13,14,15]. Molecular and pathological differences are thought to underlie these disparities between the two histological types. A comprehensive genome sequencing study by George et al. in 2015 highlighted the more aggressive molecular profile of SCLC, marked by the ubiquitous inactivation of key tumor suppressor genes, including TP53 and RB1, which drive uncontrolled proliferation and a high mitotic index [15,16,17,18]. These genetic alterations are central to the aggressive nature and poor prognosis associated with SCLC. The higher propensity for spinal metastases of SCLC, as observed in our study, is also consistent with prior research. Molecular mechanisms associated with the metastatic nature of SCLC include the overexpression of nuclear factor I B (NFIB) and MYC proto-oncogene, as well as neuroendocrine differentiation markers such as neural cell adhesion molecule (NCAM), which collectively enhance metastatic capacity [13,15]. Furthermore, Williamson et al., in 2016, described vasculogenic mimicry as a pro-metastatic mechanism in SCLC [19]. This process enables tumor cells to mimic endothelial cells, expressing endothelial markers such as vascular endothelial cadherin (VE-cadherin), which promotes angiogenesis and vascular invasion [20]. The findings from our study may also suggest that SCLC may exhibit spine-tropic features, warranting further research to elucidate the molecular underpinnings driving its high rate of metastasis to the spine.

There is currently no consensus on the optimal treatment strategy for spinal metastases originating from lung cancer. In multivariable analysis, surgery (HR: 0.46, *p* < 0.001) and radiation therapy (HR: 0.78, *p* < 0.001) were significantly associated with reduced mortality, reinforcing their central role in management. Most evidence supports the role of surgery in improving survival. Jung et al., (2021) recommended aggressive spondylectomy when feasible, highlighting its ability to reduce local recurrence and enhance survival in patients with metastatic NSCLC [21]. Similarly, studies have found that while spinal metastases from other primary cancers may not benefit significantly from surgical intervention, lung-originated metastases show improved survival following surgical treatment [22]. However, Amelot et al. (2020) reported conflicting results, finding that surgery, whether en bloc resection or decompression, did not improve survival in NSCLC patients with spinal metastases [23]. Our study supports the survival benefits of surgery alone in both NSCLC and SCLC populations with metastatic spinal disease. Notably, the higher frequency of surgery observed in the NSCLC cohort may be attributed to their comparatively better baseline health, which enables them to better tolerate and recover from complex spinal surgeries [21]. In addition, one study also showed the role of early diagnosis and treatments is associated with improved outcomes, which warrants more studies to confirm [24].

Radiation therapy remains a primary treatment modality for spinal metastases, offering effective disease control and symptom palliation [23,25]. In our study, radiation alone was the most commonly utilized modality in both NSCLC and SCLC groups, providing significant survival benefits, which is consistent with prior research [26]. While radiation modalities were evaluated for treating spinal metastases in various studies, the optimal radiation technologies for lung-originated spine metastases remain underexplored. Our findings showed that IMRT provided the best OS in the NSCLC cohort, outperforming EBRT, the current standard treatment for spinal metastases [26]. This aligns with previous studies highlighting IMRT’s ability to deliver dose-escalated, highly conformal radiation while sparing critical structures such as the spinal cord [27,28]. However, in the SCLC cohort, IMRT did not confer significant survival advantages over EBRT, suggesting a need for further research into more advanced radiation modalities tailored for this patient population. Additionally, some studies have identified SBRT as a safe and effective option for managing spinal metastases while preserving neurological function, particularly in the reirradiation setting [29,30]. However, in our study, SBRT was associated with inferior long-term survival outcomes compared to IMRT and EBRT. This finding may, in part, be attributed to the relatively small number of patients who received SBRT, potentially limiting statistical power and interpretability. Additionally, SBRT’s heightened sensitivity to spinal cord dose constraints may result in underdosing near critical structures, particularly in anatomically complex regions, thereby limiting its long-term efficacy—especially in aggressive histology such as SCLC. The reduced durability of tumor control, along with the potential for delayed toxicities such as vertebral compression fractures, radiation-induced myelopathy, and spinal instability, may also contribute to these findings. While our data demonstrate short-term survival benefits with SBRT, it is important to weigh these against the risk of late-onset complications and disease recurrence. Prior studies have shown that although SBRT offers precise local control, it may be associated with increased late toxicity, particularly at higher biologically effective doses. Moreover, recurrence patterns following SBRT can vary significantly based on tumor histology and fractionation schedules, emphasizing the importance of careful patient selection and long-term surveillance.

In terms of radiation dosage, although the clinically adopted radiation dose varied distinctively, a high dose is generally recommended to achieve satisfactory disease control. This is supported by our finding that BED 71–150 Gy was associated with a 60% reduced risk of death (HR: 0.40, *p* < 0.001). Moussazadeh et al., in 2015, reported a BED 10 of 81.6 Gy to be effective in achieving local control for bone metastases [31]. Another study also suggested a BED limit of 120 Gy as a threshold for safety [32]. Our study supported the necessity of high-dose radiation (above 70 Gy) to provide survival benefits. Moreover, the superior OS of the group treated with 71–150 Gy suggested a potentially higher threshold in managing spine metastases originating from lung cancer, particularly for NSCLC, which is known to be more radioresistant compared to SCLC [33].

Reirradiation was another significant factor in our analysis, showing an association with improved survival in patients with spinal metastases. In our analysis, reirradiation conferred a significant reduction in mortality (HR: 0.54, *p* < 0.001). This benefit likely reflects careful patient selection, improved imaging guidance, and safer delivery via IMRT and SBRT platforms. This finding is consistent with the previous literature, which identifies reirradiation as a promising option for managing local recurrence and alleviating symptoms in metastatic spinal disease [34]. Kawashiro et al. (2015) and Sasaura et al. (2020) demonstrated the safety and efficacy of IMRT and SBRT, respectively, in the reirradiation setting [28,34]. Given the histology-specific nuances in treating spinal metastases from NSCLC and SCLC, a tailored therapeutic approach is essential. Radiation therapy, particularly SBRT and SRS, is favored for its precision and association with improved early survival outcomes. Furthermore, with the advent of proton therapy, reirradiation has become a viable and increasingly utilized option, offering meaningful survival benefits in NSCLC patients with durable life expectancy.

Multiple scoring systems have been developed to predict mortality risk in patients with lung cancer and spinal metastases, serving as valuable tools for risk stratification and treatment planning. Gong et al. (2019) proposed a three-factor scoring system, identifying advanced age (≥65 years), the presence of extraosseous distant metastases, and multiple bone metastases as significant predictors of mortality [35]. Rades et al. (2020) subsequently introduced a two-factor scoring system that highlighted advanced age (≥66 years) and an Eastern Cooperative Oncology Group (ECOG) performance score ≥2 as strong predictors of poor outcomes [36]. Our study expands on these by emphasizing the critical impact of primary lung cancer histology and spine treatments on both short-term and long-term mortality risk. The SHAP analysis further demonstrated the dominant role of age, BED, and reirradiation in survival prediction. AUC scores of 0.761 at both 1- and 10-year timepoints validate the model’s robustness and suggest clinical utility for guiding individualized care planning. Advanced age is a significant prognostic factor for both short-term and long-term mortality in lung cancer patients, independent of the presence of spinal metastases, which is consistent with a recent study by Bostock et al. in 2025 [37]. This finding highlights the necessity of age-specific considerations that account for baseline health conditions in elderly populations. Furthermore, Black and Asian races were associated with an increased risk of mortality, suggesting potential racial and socioeconomic disparities that require further investigation.

Treatment-related variables also played a critical role in influencing survival outcomes. Radiation dose is a critical factor, with low dose and EBRT being predictive of short- and long-term mortality. These findings advocate for a proactive, multimodal approach with the utilization of more advanced modalities to manage spinal metastases. Future prognostic models should integrate these advanced treatment modalities and spine-specific interventions alongside demographic and molecular factors to provide a more comprehensive and personalized framework for predicting outcomes. More complicated models could also be explored to potentially improve the prediction accuracy [38,39,40].

Recent advances in imaging, such as by Lambin et al., particularly the use of radiomics and deep learning-based analysis of CT and PET/CT scans, have shown promise in enhancing lung cancer diagnosis, monitoring treatment response, and predicting patient prognosis [41]. For instance, radiomic signatures derived from high-resolution CT scans have demonstrated predictive value for tumor histology and treatment outcomes [42].

PET/CT imaging, particularly with 18F-FDG, has also been widely used to assess metabolic activity and treatment response. Moreover, artificial intelligence applications in imaging have facilitated the development of non-invasive biomarkers for risk stratification and personalized therapy planning.

### 4.1. Limitations

Despite the strengths of this study, several critical gaps remain. First, the retrospective nature of the analysis using the NCDB introduces inherent biases, such as selection bias and variability in data quality due to reliance on institutional reporting. The lack of molecular profiling limits the ability to stratify patients based on actionable genomic alterations, such as EGFR mutations, ALK rearrangements, or PIG3 mutation in NSCLC, which are associated with distinct metastatic patterns, treatment responses, and prognosis [23,39]. The lack of detailed patient baseline characteristics, such as Karnofsky, Frankel, and EQ-5D scores, limits the accuracy of adjustments for matching [23]. The absence of detailed data on tumor burden, spinal involvement severity, and number of spinal metastases precludes a more granular analysis of the impact of treatment modalities. Also, the dataset lacks clinical performance metrics such as ECOG status, Frankel grade, or imaging-confirmed levels of spinal cord compression, which are critical for treatment triage. Additionally, the study lacks functional and patient-reported data such as pain or neurological deficits, which hinders the analysis of quality-of-life outcomes. Furthermore, socioeconomic disparities observed in the study may also reflect systemic inequities, complicating the attribution of survival differences solely to treatment strategies. Finally, the study’s machine learning models, while demonstrating high predictive accuracy, were not externally validated, limiting their generalizability. Addressing these gaps in future prospective studies with comprehensive clinical, molecular, and patient-reported data, along with external validation of predictive models, is essential to enhance the robustness and applicability of the findings.

### 4.2. Future Directions

Future efforts should integrate genomic stratification (e.g., EGFR, ALK, PD-L1) to refine histology-specific treatment. Establishing prospective registries that include neurologic status, frailty scores, patient-reported outcomes, and real-time toxicity data would enhance the personalization of care. Randomized trials comparing SBRT vs. IMRT in NSCLC spinal metastases are warranted. Furthermore, external validation of AI-based models across global datasets is essential before clinical deployment.

## 5. Conclusions

Effective management of spinal metastases in NSCLC and SCLC requires a nuanced, histology-specific approach. Radiation should be prioritized for managing spinal metastases. SBRT/SRS predicts better early survival compared to other modalities, whereas SBRT/SRS and IMRT were comparable predicting better late survival. High-dose radiation should be prioritized when feasible, particularly for NSCLC patients. Reirradiation provides survival benefits to NSCLC patients with durable life expectancy, although similar benefits were not observed in SCLC. Addressing these gaps demands a proactive, multidisciplinary effort to advance therapeutic strategies, improve long-term survival, and enhance the quality of life of patients with metastatic lung cancer to the spine.

## Figures and Tables

**Table 1:** Comparison of baseline characteristics, disease features, treatment modalities, and clinical outcomes between patients with NSCLC or SCLC.

Variable	Total (N = 428,915)	NSCLC (N = 428,040)	SCLC (N = 875)	*p*-Value	Total (N = 1750)	NSCLC (N = 875)	SCLC (N = 875)	*p*-Value
	Pre-Matching				1:1 KNN Matched			
Age				<0.001				0.148
Mean ± SD (years)	61.2 ± 13.7	61.2 ± 13.7	67.2 ± 13.8		66.7 ± 13.8	66.2 ± 13.9	67.2 ± 13.8	
Sex				0.003				1.000
Male	285,673 (66.6%)	285,049 (66.6%)	624 (71.3%)		1248 (71.3%)	624 (71.3%)	624 (71.3%)	
Female	143,242 (33.4%)	142,991 (33.4%)	251 (28.7%)		502 (28.7%)	251 (28.7%)	251 (28.7%)	
Race				<0.001				0.002
White	350,881 (82.7%)	350,106 (82.7%)	775 (89.1%)		1496 (86.1%)	721 (83.1%)	775 (89.1%)	
Black	38,210 (9.0%)	38,159 (9.0%)	51 (5.9%)		126 (7.2%)	75 (8.6%)	51 (5.9%)	
Asian	11,448 (2.7%)	11,437 (2.7%)	11 (1.3%)		30 (1.7%)	19 (2.2%)	11 (1.3%)	
Hispanic	19,400 (4.6%)	19,370 (4.6%)	30 (3.4%)		69 (4.0%)	39 (4.5%)	30 (3.4%)	
Other	4221 (1.0%)	4218 (1.0%)	3 (0.3%)		17 (0.3%)	14 (0.3%)	3 (0.3%)	
N-Miss	4755	4750	5		12	7	5	
CDCC Comorbidity Scores				<0.001				1.000
0	340,059 (79.3%)	339,405 (79.3%)	654 (74.7%)		1308 (74.7%)	654 (74.7%)	654 (74.7%)	
1	64,904 (15.1%)	64,761 (15.1%)	143 (16.3%)		286 (16.3%)	143 (16.3%)	143 (16.3%)	
2	16,064 (3.7%)	16,019 (3.7%)	45 (5.1%)		90 (5.1%)	45 (5.1%)	45 (5.1%)	
3	7888 (1.8%)	7855 (1.8%)	33 (3.8%)		66 (3.8%)	33 (3.8%)	33 (3.8%)	
Tumor Size				0.520				0.268
Mean ± SD (mm)	391.25 ± 468.93	391.23 ± 468.93	401.45 ± 466.72		389.14 ± 464.98	376.83 ± 463.18	401.45 ± 466.71	
Primary Surgery				<0.001				<0.001
Yes	241,533 (56.5%)	241,162 (56.6%)	371 (42.6%)		854 (49.0%)	483 (55.4%)	371 (42.6%)	
No	185,765 (43.5%)	185,266 (43.4%)	499 (57.4%)		888 (51.0%)	389 (44.6%)	499 (57.4%)	
N-Miss	1617	1612	5		8	3	5	
Radiation Therapy				<0.001				0.728
Yes	247,156 (58.5%)	246,605 (58.5%)	551 (64.5%)		1080 (63.0%)	540 (62.6%)	540 (63.5%)	
No	175,256 (41.5%)	174,953 (41.5%)	303 (35.5%)		633 (37.0%)	322 (37.4%)	311 (36.%)	
N-Miss	6503	6482	21		37	13	24	
Chemotherapy				<0.001				<0.001
Yes	171,275 (41.0%)	170,674 (41.0%)	601 (69.8%)		920 (53.8%)	319 (37.6%)	601 (69.8%)	
NOS	15,497 (3.7%)	15,458 (3.7%)	39 (4.5%)		75 (4.4%)	36 (4.2%)	39 (4.5%)	
Single-agent	96,375 (23.1%)	96,333 (23.1%)	42 (4.9%)		639 (37.7%)	166 (19.6%)	42 (4.9%)	
Multi-agent	59,403 (14.2%)	58,883 (14.1%)	520 (60.4%)		208 (12.2%)	117 (13.8%)	520 (60.4%)	
No	246,023 (59.0%)	245,763 (59.0%)	260 (30.2%)		790 (46.2%)	530 (62.4%)	260 (30.2%)	
N-Miss	11,617	11,603	14		40	26	14	
Immunotherapy				<0.001				0.002
Yes	9699 (2.3%)	9694 (2.3%)	5 (0.6%)		26 (1.5%)	21 (2.4%)	5 (0.6%)	
No	413,885 (97.7%)	413,019 (97.7%)	866 (99.4%)		1712 (98.5%)	846 (97.6%)	866 (99.4%)	
N-Miss	5331	5327	4		12	6	4	
Clinical Outcome								
Length of Stay				0.036				0.407
Mean ± SD (days)	4.1 ± 8.9	4.1 ± 8.9	3.1 ± 7.3		3.4 ± 7.7	3.6 ± 8.0	3.1 ± 7.3	
30-Day Readmission	12,843 (3.1%)	12,819 (3.1%)	24 (2.8%)	0.693	52 (3.1%)	28 (3.3%)	24 (2.8%)	0.582
Palliative Care	9180 (2.2%)	9133 (2.2%)	47 (5.4%)	< 0.001	73 (4.2%)	26 (3.0%)	47 (5.4%)	0.012
Spine Metastasis	21,984 (5.1%)	21,865 (5.1%)	119 (13.6%)	< 0.001	216 (12.3%)	97 (11.1%)	119 (13.6%)	0.110
Mortality Rates								
1-year	64,312 (15.0%)	64,026 (15.0%)	286 (32.7%)	<0.001	458 (26.2%)	172 (19.7%)	286 (32.7%)	<0.001
5-year	144,678 (33.7%)	144,144 (33.7%)	534 (61.0%)	<0.001	933 (53.3%)	399 (45.6%)	534 (61.0%)	<0.001
10-year	167,718 (39.1%)	167,141 (39.0%)	577 (65.9%)	<0.001	1039 (59.4%)	462 (52.8%)	577 (65.9%)	<0.001
15-year	171,950 (40.1%)	171,370 (40.0%)	580 (66.3%)	<0.001	1053 (59.8%)	473 (54.1%)	580 (66.3%)	<0.001
LFU	171,972 (43.8%)	171,392 (43.7%)	580 (71.0%)	<0.001	1053 (59.8%)	473 (54.1%)	580 (66.3%)	<0.001

**Table 2:** Comparison of baseline characteristics, disease features, treatment modalities, and clinical outcomes between NSCLC or SCLC patients with spine metastases.

	Total (N = 21,984)	NSCLC (N = 21,865)	SCLC (N = 119)	*p*-Value	Total (N = 238)	NSCLC (N = 119)	SCLC (N = 119)	*p*-Value
	Pre-Matching				1:1 KNN Matched			
Age				<0.001				0.187
Mean ± SD	57.7 ± 14.4	57.6 ± 14.4	62.7 ± 11.3		61.7 ± 11.4	60.8 ± 11.5	62.7 ± 11.3	
Sex				0.120				1.000
Male	15,772 (71.7%)	15,679 (71.7%)	93 (78.2%)		186 (78.2%)	93 (78.2%)	93 (78.2%)	
Female	6212 (28.3%)	6186 (28.3%)	26 (21.8%)		52 (21.8%)	26 (21.8%)	26 (21.8%)	
Race				<0.001				0.007
White	14,669 (67.4%)	14,566 (67.3%)	103 (87.3%)		183 (77.2%)	80 (67.2%)	103 (87.3%)	
Black	2831 (13.0%)	2823 (13.0%)	8 (6.8%)		27 (11.4%)	19 (16.0%)	8 (6.8%)	
Asian	2724 (12.5%)	2723 (12.6%)	1 (0.8%)		7 (3.0%)	6 (5.0%)	1 (0.8%)	
Hispanic	1162 (5.3%)	1157 (5.3%)	5 (4.2%)		17 (7.2%)	12 (10.1%)	5 (4.2%)	
Other	387 (1.8%)	386 (1.8%)	1 (0.8%)		3 (1.3%)	2 (1.7%)	1 (0.8%)	
N-Miss	211	210	1		1	0	1	
CDCC Comorbidity Scores				0.002				1.000
0	17,793 (80.9%)	17,707 (81.0%)	86 (72.3%)		172 (72.3%)	86 (72.3%)	86 (72.3%)	
1	3059 (13.9%)	3039 (13.9%)	20 (16.8%)		40 (16.8%)	20 (16.8%)	20 (16.8%)	
2	763 (3.5%)	757 (3.5%)	6 (5.0%)		12 (5.0%)	6 (5.0%)	6 (5.0%)	
3	369 (1.7%)	362 (1.7%)	7 (5.9%)		14 (5.9%)	7 (5.9%)	7 (5.9%)	
Tumor Size				0.134				0.487
Mean ± SD (mm)	514.68 ± 481.04	514.32 ± 481.05	580.59 ± 477.05		558.92 ± 479.81	537.24 ± 483.60	580.59 ± 477.05	
Treatment Status of Spine Metastasis				<0.001				0.008
Treated	19,194 (87.3%)	19,110 (87.4%)	84 (70.6%)		185 (77.7%)	101 (84.9%)	84 (70.6%)	
Conservative	2790 (12.7%)	2755 (12.6%)	35 (29.4%)		53 (22.3%)	18 (15.1%)	35 (29.4%)	
Treatment to Spine Metastases				<0.001				<0.001
Surgery alone	5993 (31.2%)	5987 (31.3%)	6 (7.1%)		35 (18.9%)	29 (28.7%)	6 (7.1%)	
Radiation alone	13,011 (67.8%)	12,935 (67.7%)	76 (90.5%)		145 (78.4%)	69 (68.3%)	76 (90.5%)	
Surgery + radiation	190 (1.0%)	188 (1.0%)	2 (2.4%)		5 (2.7%)	3 (3.0%)	2 (2.4%)	
N-Miss	2790	2755	35		53	18	35	
Reirradiation Status				0.252				0.798
Yes	3425 (26.2%)	3409 (26.2%)	16 (20.5%)		32 (21.3%)	16 (22.2%)	16 (20.5%)	
No	9645 (73.8%)	9583 (73.8%)	62 (79.5%)		118 (78.7%)	56 (77.8%)	62 (79.5%)	
N-Miss	8914	8873	41		88	47	41	
Beam Technology of Spinal Radiation				0.023				0.064
EBRT	5555 (42.5%)	5514 (42.5%)	41 (52.6%)		72 (48.0%)	31 (43.1%)	41 (52.6%)	
IMRT	7110 (54.4%)	7078 (54.5%)	32 (41.0%)		73 (48.7%)	41 (56.9%)	32 (41.0%)	
Conformal (3-D) therapy	309 (2.4%)	306 (2.4%)	3 (3.8%)		3 (2.0%)	0 (0.0%)	3 (3.8%)	
SBRT/SRS	86 (0.7%)	84 (0.6%)	2 (2.6%)		2 (1.3%)	0 (0.0%)	2 (2.6%)	
N-Miss	8924	8883	41		88	47	41	
Biologically Effective Dose (BED)				0.019				0.083
Mean ± SD (Gy)	70.6 ± 44.8	70.7 ± 44.9	57.9 ± 25.5		62.433 (29.548)	66.350 (32.268)	57.882 (25.521)	
BED strata				<0.001				0.243
Below 70 Gy	5601 (38.1%)	5560 (38.0%)	41 (60.3%)		79 (53.7%)	38 (48.1%)	41 (60.3%)	
71–150 Gy	8990 (61.1%)	8963 (61.2%)	27 (39.7%)		67 (45.6%)	40 (50.6%)	27 (39.7%)	
Above 150 Gy	114 (0.8%)	114 (0.8%)	0 (0.0%)		1 (0.7%)	1 (1.3%)	0 (0.0%)	
N-Miss	7279	7228	51		91	40	51	
Chemotherapy				<0.001				0.026
Yes	15,690 (72.7%)	15,587 (72.6%)	103 (87.3%)		192 (81.7%)	89 (76.1%)	103 (87.3%)	
No	5905 (27.3%)	5890 (27.4%)	15 (12.7%)		43 (18.3%)	28 (23.9%)	15 (12.7%)	
N-Miss	389	388	1		3	2	1	
Clinical Outcome								
Length of Stay				0.438				0.728
Mean ± SD (days)	5.7 ± 11.1	5.7 ± 11.1	3.8 ± 10.1		3.4 ± 8.1	2.9 ± 5.1	3.8 ± 10.1	
30-Day Readmission Rate	554 (2.6%)	551 (2.6%)	3 (2.6%)	0.992	4 (1.7%)	1 (0.9%)	3 (2.6%)	0.322
Palliative Care Rate	1503 (6.8%)	1488 (6.8%)	15 (12.6%)	0.012	22 (9.2%)	7 (5.9%)	15 (12.6%)	0.073
Mortality Rates								
1-year	4198 (19.1%)	4144 (19.0%)	54 (45.4%)	<0.001	87 (36.6%)	33 (27.7%)	54 (45.4%)	0.005
5-year	8520 (38.8%)	8437 (38.6%)	83 (69.7%)	<0.001	136 (57.1%)	53 (44.5%)	83 (69.7%)	<0.001
10-year	9566 (43.5%)	9477 (43.3%)	89 (74.8%)	<0.001	154 (64.7%)	65 (54.6%)	89 (74.8%)	0.001
15-year	9771 (44.4%)	9682 (44.3%)	89 (74.8%)	<0.001	157 (66.0%)	68 (57.1%)	89 (74.8%)	0.004
LFU	9771 (49.2%)	9682 (49.0%)	89 (82.4%)	<0.001	157 (71.0%)	68 (60.2%)	89 (82.4%)	<0.001

## Data Availability

The data presented in this study are available on reasonable request. The data are not publicly available due to privacy/ethical restrictions.

## Abbreviations

The following abbreviations are used in this manuscript:

NCDB	National Cancer Database
NSCLC	Non-small cell lung cancer
SCLC	Small cell lung cancer
OS	Overall survival
RT	Radiation therapy
SBRT	Stereotactic body radiation therapy
SRS	Stereotactic radiosurgery
BED	Biologically effective dose
CDCC	Charlson–Deyo Comorbidity Classification
LOS	Length of stay
KM	Kaplan–Meier
AUC	Area under the curve
HIPAA	Health Insurance Portability and Accountability Act
STROBE	Strengthening the Reporting of Observational Studies in Epidemiology
SMOTE	Synthetic Minority Over-sampling Technique
SHAP	SHapley Additive exPlanations
ECOG	Eastern Cooperative Oncology Group
